# Recommendation of Electricity for the Cure of the Cataract; Illustrated by a Case

**Published:** 1786

**Authors:** Charles Kite

**Affiliations:** Member of the Corporation of Surgeons of London, and Surgeon at Gravesend in Kent


### VI. *Recommendation of Electricity for the Cure of the Cataract; illustrated by a Case. By Mr.* Charles Kite, *Member of the Corporation of Surgeons of London, and Surgeon at Gravesend in Kent.*

MRS. B. of Gravesend, a widow, aged sixty, much subject to hypochondriac affection, was, in May, 1785, without any apparent cause, seized with a pain in the fore part of her head, and shortly after with a considerable dimness of sight: in a few days these symptoms became so urgent, that my assistance was requested.

I was then informed the pain occupied the entire fore part of the head, both internally and externally, but that it was more particularly severe in the space between the temples: it was said to be neither burning, pricking, nor pulsatory, but dull, heavy, and constant, neither changing its place, nor becoming intermittent, or even remittent; it was the same in an horizontal and in an erect position, and it did not seem materially increased either by coughing, sneezing, or a long-continued inspiration.

Her pulse was natural, both as to strength and quickness. Her tongue was moist, and

ſomewhat white. There was no giddineſs, or tendency to delirium, and all her ſenſes, that of ſeeing excepted, were perfectly clear.

Her appetite and ſleep had almoſt forſaken her; her ſpirits were extremely low, and her countenance carried indubitable marks of the anxiety of her mind.

On examining her eyes, I found the cryſtalline humours changed to a pearl colour, and obſerved two or three ſpecks, the remains of former inflammations, on the cornea of one eye; one of theſe ſpecks was ſituated immediately before the pupil, ſo that it prevented my having a perfect view of the cryſtalline; and as in ſome meaſure it excluded the rays of light from falling on the retina, it probably occaſioned the iris to have very little motion. Whether this, however, was really the reaſon or not, I will not determine; but the fact is, the pupil of this eye was much ſmaller than that of the other, and it had likewiſe leſs motion.

She very readily perceived the light, and was capable of diſtinguiſhing bright and luminous objects; but the darker colours made very little impreſſion upon her; and what rather ſurpriſed me was, that ſhe appeared to be nearly as ſenſible of the light, &c. with that eye which had ſpecks before the pupil, and which had little or no motion in it, as with the other, which was perfectly free from thoſe circumſtances.

A large bliſter was applied to the neck, ſome æthereal ſpirit was frequently held to her forehead, and ſhe was directed to take ſome gentle phyſic. By theſe means the pain in the head was very much alleviated, and, in a few days, it entirely ceaſed. The opacity of the cryſtalline humours, however, did not abate, and her blindneſs, if there was any alteration, ſeemed rather to increaſe. The depreſſion of the humour was recommended; but ſhe perſiſted in the determination of ever remaining in her preſent wretched ſituation, rather than ſubmit to that operation.

That nothing might be omitted from which even the moſt diſtant probability of ſucceſs might ariſe, I determined to try the effects of electricity, thoroughly perſuaded of the total inefficacy of every other remedy; and indeed, from this wonderful agent, my expectations were not very ſanguine.

The electric fluid, therefore, was, by means of a wooden point, drawn from the cornea, while ſhe ſat on the inſulated chair daily for the ſpace of a month. The operation each time was continued about a quarter of an hour. At the expiration of the month I did not perceive the opacity of the humours was at all diminiſhed, nor did my patient think that her ſight was in any degree altered for the better. I was unwilling, however, to relinquiſh the only plan from which I could reaſonably expect to derive any benefit, and therefore reſolved to direct ſmall ſparks through the diſeaſed humours; this was done about a week, and then very ſmall ſhocks, from a jar containing about an hundred inches of coated ſurface, were ſubſtituted in their place. The gentleſt ſhock, however, that could be given from this was ſo powerful, and irritated her ſo much, that a ſmall vial, with no more than twelve inches, was uſed in lieu of the larger one: forty or fifty ſhocks from this, of not more than one tenth of an inch in length, were daily directed through each eye, and theſe ſhe bore tolerably well; but if either their number or ſtrength were materially increaſed, it occaſioned a ſevere pain in the head.

Soon after this courſe had commenced, ſhe began to ſee ſomewhat diſtinctly; and when it had had been continued a month, ſhe was ſo much benefited as to be able to read, at one time, ten or twelve pages of very ſmall print, and to hem an handkerchief in a tolerable manner, equally well indeed, I was informed, as ſhe had done two years before.

The cryſtalline humours were now almoſt, if not entirely, as clear as is uſual in people of her age, and, in ſhort, ſhe was altogether infinitely better than my moſt ſanguine expectations could have allowed me to hope.

I mentioned, in the beginning of this account, that my patient was, at times, ſubject to hypochondriac affections: I am extremely concerned to have occaſion to add, that, about this time, ſhe was again attacked with that complaint, which now reſembled the diſeaſe treated of by Sauvages under the title of Hypochondriaſis Melancholica.

This unfortunate occurrence cloſed our proceedings, and, with that, every idea of farther recovery vaniſhed.

As in her previous attacks, if no real cauſe did occur, ſome imaginary evil was pitched upon as the ſource of her mental ſufferings, ſo, in the preſent inſtance, the failure of her ſight was fixed on as the reaſon of this, although it was extremely evident both to myſelf and her friends, that the diſeaſe did not at all depend upon the cauſe to which it was attributed.

I was firſt induced to make trial of electricity in this caſe, by reflecting on the great advantage I had in many inſtances received from its application in obſtructions of various parts of the body, but more particularly in inflammations of the external coats of the eyes; and that whether the inflammation was mild or ſevere, recent, or of long ſtanding: in ſhort, whether it was a truly acute inflammation, or merely a relaxation and over-diſtention of the veſſels in conſequence of inflammation, I do not recollect a ſingle inſtance in which it did not effectually anſwer my expectations.

The real cauſes of theſe opacities are probably unknown; but when we recollect that the capſula of the cryſtalline humour is ſupplied with capillary veſſels from ſome of the ſmall ramifications of the ocular artery, and that veſſels have been ſeen running from the capſula into the body of the humour itſelf, we can eaſily conceive that inflammation and obſtruction may, upon the application of certain exciting cauſes, juſt as readily occur in theſe veſſels as in thoſe of the cornea, ſclerotica, or any othe other part. Now, as I before mentioned, that in inflammations and obſtructions of the external membranes of the eye, I had applied electricity with greater advantage than any other means; and, as I conceived it probable there was ſome analogy between thoſe diſeaſes and the caſe under conſideration, I was induced to employ the ſame remedy. And although electricity in the above caſe did not effect a cure, yet the advantages derived from it were ſo very evident, and ſo infinitely ſuperior to what I ever ſaw produced by any other means, that I have no doubt but a complete cure would have been accompliſhed, if our operations had not been prematurely ſtopped; and I am the more diſpoſed to be of this opinion, ſince I have read Dr. Knox's account, in the ninth volume of the Medical Commentaries, of cataracts in both eyes removed by electricity. It does not appear, however, that his patient was affected in ſo great a degree as the ſubject of the caſe I have related; but I am much inclined to think, that if the remedy had been more powerfully employed, the cure would have been conſiderably expedited.

A variety of medicines have, at different times, been recommended for the removal of cataracts; but now that the nature and ſeat of the diſeaſe are thoroughly aſcertained, very little confidence is placed in any of them, even in the moſt recent caſes, and under the moſt favourable circumſtances. Mercury is, I believe, allowed to ſtand at the head of theſe remedies; and I have once or twice ſeen good effects ariſe from its uſe, in caſes which were ſuppoſed to be incipient cataracts. I have likewiſe, in more inſtances than one, ſeen the ſame medicine exhibited in caſes of ſome ſtanding, and where the opacity was nearly complete, but it was never attended with any advantage.

The inefficacy of theſe remedies has induced practitioners to lay them aſide, and rely entirely for the cure on the removal of the humour.

It is with the view of calling the attention of medical gentlemen to a new remedy that the preſent caſe is laid before them; and as it is a ſubject extremely intereſting to humanity, comprehending nothing leſs than the happineſs or miſery of many unfortunate individuals, I have not a doubt but it will receive, what it appears in an eminent degree to merit, a fair and candid trial: it is that which will determine its efficacy, and by that it muſt ſtand or fall. If, as we have reaſon to expect, it ſhould, by experience, perience, be found to prove fuccefsful, the fight will be more perfect than when either depreffion or extraction has taken place; as in the former cafe, the lens is merely rendered tranfparent, whereas in the latter it is entirely deftroyed, and confequently the patient cannot have a diftinct vifion without the affiftance of a convex glafs, as a fubftitute for the humour which is loft. If, on the contrary, it fhould not be found to fucceed, we have the fatisfaction of knowing that if it be judicioufly applied, it cannot produce the leaft inconvenience.

I cannot conclude without obferving, that it appears to me, the more powerfully electricity is applied, provided it does not irritate too much, or occafion too great uneafinefs, the more likely will it be to haften the cure. No particular degree of force can, with propriety, be fixed on as a ftandard; for a weak, delicate, irritable woman will be as much affected by a moderate fpark, as a hearty, ftrong, robuft man will by a fmart fhock. Its ftrength, like the dofe of an active medicine, muft be accommodated to the habit and conftitution of the patient.

By beginning moderately, and increafing its ftrength gradually, we fhall be certain to avoid doing any mifchief, and fhall readily be able to afcertain the bounds, beyond which we muft not proceed. I would therefore advife, that, at firft, a ftrong current of electrical aura be directed through the difeafed parts: this may be effected (after placing the patient in an infulated chair) by running a pin through the hair, &c. fo as to be in contact with the hind part of the head, immediately oppofite the eye we intend to operate upon, and connecting it with the cufhion; at the fame time, a director, with a wooden point communicating with the prime conductor of a powerful machine, is to be held at a fmall diftance from the eye, and, on putting the cylinder in motion, the fluid will pafs from one point to the other, and confequently through the difeafed humours.

When this has been repeated ten or twelve times, fmall fparks fhould be fubftituted in its place. Thefe may very advantageoufly be given, by placing the knob of a director, connected with the electrometer, fo as to touch the moft prominent part of the eye, while the pin, connected with the negative conductor, is fixed as in the laft method; and in this manner the ftrength of the fparks may be determined the fame as fhocks.

As

As soon as it is thought proper to have recourse to shocks, it will be necessary, in addition to the last-mentioned plan, to suspend a small coated vial from the conductor, and to connect the pin with the outside of the vial instead of the cushion.

There is still another mode of applying our remedy, and it is from this modification of the electric fire I entertain the greatest expectations, as it appears peculiarly adapted to answer our present purpose. It conveys a sensation between a spark and a shock, and is produced by means of a tube inserted into the vial, the lower end of which is coated, and in contact with a wire which passes through a ball on the top of the tube. As no particular management is required for this, farther than that already recommended for the application of the shocks, it will be unnecessary to say any thing more upon the subject; and indeed I should think it proper to apologise for having already taken up so much time, if I did not think these few hints might save those some trouble who have not previously been in the habit of making the experiments.

*Gravesend,*

February 22, 1786.

